# Dicephalus parapagus conjoined twins discordant for anencephaly: a case report

**DOI:** 10.1186/1752-1947-4-38

**Published:** 2010-02-05

**Authors:** Usang E Usang, Babatunde J Olasode, Ayi E Archibong, Jacob J Udo, Diana-Abasi U Eduwem

**Affiliations:** 1 University of Calabar/University of Calabar Teaching Hospital, Calabar, Cross River State, Nigeria; 2 Obafemi Awolowo University/Obafemi Awolowo University Teaching Hospitals Complex, Ile Ife, Osun State, Nigeria

## Abstract

**Introduction:**

Cases of conjoined twins occur so rarely that it is important to learn as much as possible from each case.

**Case presentation:**

We present a case of 9-hour-old, female, Nigerian dicephalus parapagus conjoined twins discordant for anencephaly diagnosed only after the birth of the twins. The anencephalic twin was stillborn while the normal one died within 9 hours of birth from cardiopulmonary failure.

**Conclusion:**

Many congenital defects of interest can now be detected before birth. A severe lesion such as that found in our index case, which is incompatible with postnatal life, requires counselling. If detected early enough during a properly monitored antenatal care, it may indicate termination of pregnancy.

## Introduction

Conjoined twinning is a rare phenomenon, occurring in 1 in 50,000 to 100,000. However, since 60% are stillborn or die shortly after, the true incidence is around 1 in 200,000 live births [1]. A rarer form of conjoined twinning is the dicephalus parapagus twins discordant for anencephaly in which the laterally united babies have two heads in one trunk. One of the twins has no cranium or brain tissue, but both have upper limbs and two lower limbs. Whereas the incidence of conjoined twinning in our country is unknown, there have been previous reports from Nigeria [2].

We recently encountered live dicephalus parapagus conjoined twins discordant for anencephaly who survived for 9 hours after delivery.

## Case presentation

9-hour-old female conjoined twins with one torso and two heads were brought into the sick babies unit (SBU) by a 25-year-old Nigerian mother of the Ekoi tribe in Cross Rivers State who just had her first delivery. She had limited antenatal care (ANC) in a primary health centre where no antenatal ultrasonography had been carried out. The pregnancy, which was carried to term, was characterized by regular use of an herbal enema from the onset and polyhydramnios. The delivery had been completed vaginally at home without any obstruction to labour. The normal head presented first. Only the normal twin cried after several minutes of stimulation. The combined weight of the conjoined twins at the time of admission was 2.85 kg.

**Figure 1:**
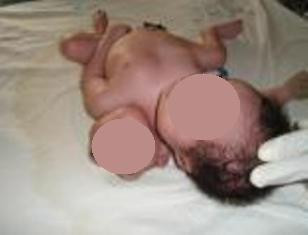
**The conjoined twins with discordant heads**.

Clinical examination revealed two discordant heads (Figure 1). The normal and anencephalic heads had an occipito-frontal circumference of 34 cm and 24 cm, respectively. There was a single thorax with two neurologically independent upper limbs, single abdomen, one complement of genitalia and an anus as well as two neurologically independent lower limbs.

At presentation in the SBU, the anencephalic twin was unresponsive to painful stimulus with dilated and unreactive pupils. An orogastric tube was inserted that ended in the neck of the twin.

The normal twin remained stable for a short while but soon experienced repeated apneic attacks with cyanosed extremities. Though prompt resuscitative measures were taken, the twin died within 9 hours of birth from cardiopulmonary failure. As a result of their unstable condition and the short duration of life, thorough investigation of the twins was not possible.

### Post-mortem Babygram findings

**Figure 2:**
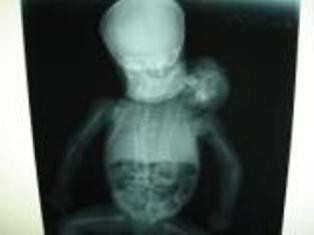
**A plain X-ray of the twins showing two separate spines**.

Post-mortem plain X-ray findings showed a fully developed cranium with normal facial structures continuous with the main body. The second head was devoid of a cranium. Each cranium was connected via a separate spine that terminated abruptly at the fifth lumber vertebrae with no evidence of any sacral component (Figure 2). The ribs on the medial side of each twin were fused with each other creating 12 instead of 24 posterior ribs, but the other ribs had not fused. Each upper limb and clavicle appeared borne by the twin on that side.

The lungs and heart were not demonstrable but the single pelvis and lower limbs are clearly defined.

### Autopsy findings

The head with normal calvaria contained a well-formed brain whereas the anencephalic head had no forebrain. Two complements of neck organs and two vertebral columns were demonstrable. The right trachea continued to a right-sided pair of normal lungs while the left trachea joined a pair of collapsed and hypoplastic lungs. A single intestinal tract opened to the exterior as a well-formed anal canal. The other abdominal and pelvic organs were not duplicated.

**Figure 3:**
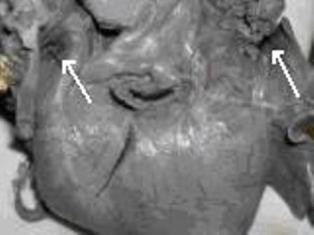
**The twins' single heart with paired great vessels (arrows)**.

Two pairs of great vessels (Figure 3; arrows), two aortic and two superior vena cavae entered the single heart. There were two atria, two rudimentary auricular appendages and two ventricles.

## Discussion

Prenatally diagnosed dicephalus conjoined twins discordant for anencephaly has been reported but is rare [3]. It is also rare for such an anomaly to escape antepartum diagnosis and only present at birth, as in the case of our patients, with the current antenatal screening tests carried out in developed countries.

The relationship between conjoined twinning and anencephaly is not well understood. However, it has been observed that the incidence of congenital malformations is significantly increased in conjoined twinning, probably due to the later incomplete fission of the monozygotic embryo during embryogenesis (fission theory) or due to secondary union of two originally separate monovular embryonic discs (fusion theory) [4]. For this reason, it is claimed that the same aetiological factor could be responsible for both the conjoining process and congenital malformations [5]. Consequently, there is failure of the neural tube at the cranial end during the fourth week of development [6] causing the forebrain primordium to be abnormal and the development of the calvaria to be defective. This gives rise to anencephaly which is a fatal disorder. While 50% of cases result in fetal demise, the rest die at birth or shortly thereafter as was the case with our discordant conjoined twins. This disorder is also associated with a high risk of preterm delivery before 32 weeks due to the development of polyhydramnios [7], possibly due to the fetuses lacking the neural control necessary for swallowing amniotic fluid. This is probably responsible for the few reported cases of the anomaly in the literature.

Spitz [8], in a study of conjoined twins, concluded that one third of those born alive have severe defects for which surgery is not possible. Similarly, Golladay *et al*. [9] observed that surgical separation is feasible only when the upper portions of the trunks are sufficiently separate to provide a stable rib cage for each infant. We agree with these authors because the clinical, radiologic and morbid study of our twins showed that separation was impossible. In the case of monozygotic twins discordant for anencephaly, selective termination by occlusion of the umbilical vessels of the abnormal fetus [10] would be the optimal management for the future. This prevents transplacental passage of injurious agents through the common placenta to the normal co-twin, which would occur if this selective termination was achieved by intracardiac injection of potassium chloride [11].

However, when twins are conjoined, as in the case of our patients, they not only share one placenta but have a single umbilical cord through which umbilical vessels are shared [12], therefore selective termination is impossible. We therefore agree with Owolabi *et al*. [13] that termination of pregnancy should be advised in cases where dicephalic twins are detected early *in utero*, especially if there is discordance for anencephaly as in the case of our patients.

Screening the serum of pregnant women at 16 to 18 weeks' gestation for alpha-fetoprotein can result in the detection of about 80% of fetuses with anencephaly and other neural tube defects [14]. If a woman has a high alpha-fetoprotein level, ultrasonography is performed to determine whether an abnormality is present. With the advent of high resolution ultrasonography, conjoined twins can be picked up as early as the 8th week of gestation and with fetal echocardiography as well as ultra fast magnetic resonance imaging, evaluated for possibility of postnatal survival [15].

However, most of these facilities are lacking in many of our country's institutions. Moreover, many of the patients do not register for ANC due to poverty and being ill-informed, as in our index case. As a result, prenatal diagnosis of congenital anomalies is unlikely in our region.

## Conclusion

This case emphasizes the need for ANC with prenatal ultrasound monitoring of high-risk pregnancies in order to determine the nature of the perinatal management required. When serious malformations that are incompatible with postnatal life are diagnosed early enough, the family has the option of terminating the pregnancy. Therefore, there is a need to improve our health care delivery system to make such services available and accessible to all our pregnant women. Similarly, it is important to educate the women and their spouses on the need for proper ANC.

## Abbreviations

ANC: antenatal care; SBU: sick babies unit.

## Consent

Written informed consent was obtained from the parents for the publication of this case report and any accompanying images. A copy of the written consent is available for review by the journal's Editor-in-Chief.

## Competing interests

The authors declare that they have no competing interests.

## Authors' contributions

UEU drafted the manuscript. BJO performed the autopsy and also joined in drafting the manuscript. AEA and JJU supervised treatment and drafting of the manuscript. DUE reported on the post-mortem radiologic findings and helped to draft the manuscript. All authors have read and approved the final manuscript.
